# Hibernation telomere dynamics in a shifting climate: insights from wild greater horseshoe bats

**DOI:** 10.1098/rspb.2023.1589

**Published:** 2023-10-11

**Authors:** Megan L. Power, Roger D. Ransome, Sébastien Riquier, Luke Romaine, Gareth Jones, Emma C. Teeling

**Affiliations:** ^1^ School of Biology and Environmental Science, Science Centre West, University College Dublin, Belfield, Dublin 4, Republic of Ireland; ^2^ School of Biological Sciences, University of Bristol, Life Sciences Building, 24 Tyndall Avenue, Bristol BS8 1TQ, UK

**Keywords:** telomeres, torpor, ageing, greater horseshoe bat, weather, telomerase

## Abstract

Hibernation is linked with various hypotheses to explain the extended lifespan of hibernating mammals compared with their non-hibernating counterparts. Studies on telomeres, markers of ageing and somatic maintenance, suggest telomere shortening slows during hibernation, and lengthening may reflect self-maintenance with favourable conditions. Bats in temperate zones adjust body temperatures during winter torpor to conserve energy and exploit mild conditions for foraging. Climate change may impact the hibernation cycle of bats, but more research is needed regarding the role of telomeres in understanding their response to a changing climate. Here, relative telomere length (rTL) was measured in the long-lived greater horseshoe bat *Rhinolophus ferrumequinum* (*n* = 223 individuals) over three winters, considering climatic conditions. Cross-sectional analyses revealed between-individual variation in rTL with a strong year effect, likely linked to varying weather conditions and foraging success. Additionally, within-individual increases of rTL occurred in 51% of consecutive measurements, with evidence of increasing telomerase expression during hibernation in this species. These findings highlight the beneficial effects of hibernation on telomeres and potential consequences of changing climatic conditions for long-lived temperate bats. Understanding the interplay between hibernation, telomeres, and climate can provide insights into the adaptive capacity and survival of bat populations facing environmental challenges.

## 1. Introduction

To maximize survival in seasonal environments, some mammals enter hibernation in the winter. Mammalian hibernation involves prolonged torpor characterized by a reduction in body temperature, metabolic rates and other physiological functions [1–3], conferring significant energy savings. Torpor is associated with longer lifespans [4–6], with little consensus on the different hypotheses behind this phenomenon (e.g. investment in self-maintenance [7], reduction of metabolic processes [8,9]). Additionally, many hibernators interrupt multiday torpor bouts with periodic arousals to normal body temperature, which although brief, are energetically expensive and can account for up to 90% of overwinter energy expenditure [1,10]. While the reasons for arousals during torpor are unclear, they may be associated with the need for water, foraging, mating, or movement between hibernacula [11–13]. Frequent and/or prolonged arousal activity can cause rapid fat depletion [14], with more frequent arousals at higher ambient temperatures [15,16]. As global annual temperatures are rising with greater warming at higher latitudes [17], temperate and arctic hibernators are susceptible to negative effects of increased arousals [18]. Further, long-lived species are vulnerable to climate change as they struggle to adapt to rapidly changing climatic conditions [19].

Telomeres, conserved nucleoprotein repeats (TTAGGG_n_) that cap the end of eukaryotic chromosomes, are biomarkers of somatic maintenance [20,21]. Telomeres shorten due to incomplete replication during cell division [22]. They are also thought to shorten through single-strand DNA damage caused by reactive oxygen species (ROS) [23], though evidence for this relationship is mixed [24–26]. Yet, losses can be repaired by other mechanisms, such as extension at the 5′ end of the chromosome via the enzyme telomerase [27], although telomerase is typically repressed in somatic tissues [28]. Telomere dynamics in hibernators have shown arousal frequency is positively correlated with accelerated telomere shortening and negatively correlated with time spent torpid [29–33]. Maintenance of telomere length (TL) during torpor is not surprising given that cell division is severely depressed at low temperatures during hibernation [34,35], with telomere shortening during arousal periods likely occurring due to increased mitotic activity and an increase in ROS [34,36]. Telomere lengthening may reflect investment in self-maintenance when conditions are favourable [29,36,37], suggesting telomeres can be used as biomarkers of an individual's state. The implications of telomere lengthening, when it occurs, and how it affects natural populations that hibernate under a variety of climatic and ecological conditions is still poorly understood.

Bats (order Chiroptera), are extremely long-lived for their body size, have low reproductive rates, and show few signs of senescence [6,38]. Bat species in temperate zones allow their body temperature to fall to ambient levels during winter torpor [39]. While most bats accumulate fat reserves prior to hibernation, bats have a limited capacity for fat storage in order to maintain the ability to fly [40]. Many insectivorous temperate-zone bats forage in the winter, especially in maritime climates [11], as aerial insects fly during mild winter weather [41]. Therefore, hibernating bats must balance the physiological costs of being torpid against the energetic costs of arousals [13]. Bats in temperate zones typically spend hibernation in underground sites (hibernacula) where large air volumes and cold substrates can increase heat loss [26]. Predicted increases in the frequency of warm winters and earlier springs due to climate change may result in bats having shorter hibernation seasons, increased arousal rates and emerging from hibernation earlier [42]. Furthermore, their reproductive cycle is closely tied to their hibernation season [43], with changes in ambient temperature during hibernation potentially impacting reproductive success and survival. However, there is a need for more studies in bats on the physiological and molecular responses to a changing climate [44], with telomeres offering a promising marker to understand such responses. Patterns of telomere dynamics in bats have only recently been uncovered, with cross-sectional analyses suggesting slow age-dependent attrition in two bat species (the greater horseshoe bat (*Rhinolophus ferrumequinum*) and the common bent-wing bat (*Miniopterus schreibersii*)) and no evidence of shortening with age in two species of *Myotis* [45]. In the greater mouse-eared bat (*Myotis myotis*), more favourable temperature conditions suggested lengthening of telomeres [46]. Yet in bats, the influence of hibernation on responses to climate is poorly understood with no knowledge on telomere dynamics in hibernating bats.

The *R. ferrumequinum* population in Gloucestershire, UK has been extensively studied since the late 1950s [11,12,47–52]. Annual autumn/winter surveys show that most individuals hibernate in caves and mines within a 25 km radius of the maternity roost [49], though some can travel up to 40 km to hibernacula [53]. Hibernation occurs from October to May in underground sites [47]. *Rhinolophus ferrumequinum* arouses frequently (typically at dusk) during torpor with the number of arousals increasing at warmer temperatures [47,54], and torpor bouts ranging from 0 to 12 days [46]. While there are probably multiple reasons for arousal, foraging occurs during many arousals [12,47], in contrast to other hibernators at cold climates that show little or no foraging during hibernation [55]. The close synchronization of arousals with dusk by bats with the lowest body reserves [54] supports successful winter foraging by bats, and amounts eaten overnight in the winter can sometimes rival summer consumption [12]. Aerial insects become active and more abundant in temperatures above 10°C [41]. However, wet conditions can reduce or prevent successful foraging [51,52]. Arousal frequencies are usually high in early hibernation [47] and normally reach their lowest levels during mid-winter where time spent in torpor is at its greatest [56]. Initial cross-sectional analyses of *R. ferrumequinum* telomeres showed shortening with age [45], though it is not known how patterns vary across the life cycle. As this species is long-lived given their small size (maximum lifespan: 30.5 years, longevity quotient (observed longevity/expected longevity): 4.89 [45,50]), torpor could provide maintenance periods allowing recovery from more stressful events (e.g. early-life, reproduction) given current knowledge on telomere dynamics in other hibernators.

Here, we tested if torpor confers a protective effect on telomeres while also considering climatic conditions. We measured relative telomere length (rTL) at different time points during hibernation (from early hibernation, throughout the hibernation season to emergence and post-hibernation) in wild greater horseshoe bats (*R. ferrumequinum*). We predicted that rTL would show little or no change within hibernation periods. In addition, we tested if weather conditions (specifically temperature and precipitation) influenced variation in rTL. We hypothesized that warm and dry conditions lead to shorter telomeres given increases in arousal frequency during warmer weather.

## 2. Material and methods

### (a) Study population

This study focuses on hibernacula surrounding a long-term maternity colony (approx. 180–240 individuals) of *R. ferrumequinum* at Woodchester Mansion, Gloucestershire (51°43′ N, 2°18′ W). All bats born at the maternity colony are ringed soon after birth so exact age is known for ringed individuals [49]. *Rhinolophus ferrumequinum* individuals were captured using hand nets during annual hibernacula surveys from October to May in the following winters: 2015/2016, 2017/2018 and 2019/2020. All individuals were weighed to an accuracy of ±0.1 g using a precision weight balance, sexed and aged if ringed. Forearm length (radius) measurements were also taken to an accuracy of ±0.1 mm and used to estimate body condition. Bats not born at the maternity colony were ringed with stainless steel rings (4.2 mm alloy aluminium bat rings, Porzana, UK). Sub-adult *R. ferrumequinum* and adult bats were aged from their sexual condition, pelage colour and state of the epiphyseal–diaphyseal joints in the finger bones [54,57]. Newly ringed adult bats were assigned the minimum age of sexual maturity in this species (i.e. approx. 2 years old [57]).

While TL is typically measured from blood, extracting sufficient volumes from bats during hibernation is not feasible [58] and buccal swab rTL measurements in bats are affected by storage time [59]. Wing tissue was chosen as it allows for repeated, non-lethal sampling, has strong repeatability within individuals [58] and is little affected by storage time (see below). Wing biopsies were taken using a sterile 3 mm wing biopsy punch and immediately transferred to individually labelled tubes of silica beads at room temperature (*n* = 399 samples, *N* = 227 individuals). Samples were stored at −20°C between 2 and 838 days (median: 114 days) prior to DNA extraction.

Samples were grouped into five sampling sessions across the hibernation period (October–May) to account for dispersal of individuals across different hibernation sites and multiple sampling trips across short time periods to maximize the number of samples collected. Each session spanned two to three weeks and are classed as: 1 (late October–mid November; *n* = 118 samples, *N* = 111 individuals), 2 (late January–early February; *n* = 113, *N* = 110), 3 (late March–early April; *n* = 88, *N* = 82), 4 (late April–early May; *n* = 40, *N* = 40) and 5 (late May; *n* = 39, *N* = 39). Arousals are normally frequent in early and late hibernation, especially in April/May depending on weather conditions which can be seen with lower sample sizes in later sampling sessions. Lowest arousal frequencies occur in mid-winter [46].

### (b) Molecular and relative telomere length analyses

Genomic DNA was extracted from wing biopsies using membrane filter kits; Promega Wizard SV DNA Extraction Kit (catalogue no. A2371) or the Qiagen DNeasy Blood and Tissue Kit (Qiagen, CA, USA). Extractions carried out with the Promega kit were partially automated using a Hamilton STAR Deck liquid handling robot but otherwise followed the manufacturer's instructions. DNA integrity from a subset of samples (22%) was confirmed using electrophoresis on a 1% agarose gel (integrity score less than 3 as per [60]). DNA yield and purity were quantified using a BioDrop µLite spectrophotometer (BioChrom, UK). DNA samples with a 260/280 absorption ratio (a measure of protein and salt contamination) of less than 1.7 were excluded from further analyses. DNA yield and purity did not differ across sampling session (Kruskal–Wallis, DNA yield: *H*_4_ = 6.880, *p* = 0.142; DNA purity: *H*_4_ = 3.624, *p* = 0.459). Extractions failed for six samples and were excluded, leaving 393 samples from 225 individuals. DNA extractions were stored at −80°C until telomere analysis (2–167 days (median: 21 days)). rTL was measured by real-time quantitative PCR as the concentration of telomeric DNA relative to the mammalian brain-derived neurotrophic factor (BDNF) that is constant in number and optimized for use in bats [45]. Samples were randomly allocated onto qPCR plates along with samples collected as part of other projects in this long-term study. Reaction composition and conditions were as detailed in previous studies, with telomere and single copy gene (SCG) reactions carried out on different qPCR plates due to differences in optimum annealing temperatures [45,46,58] (see electronic supplementary material for further details on qPCR method and quality control metrics).

### (c) Statistical analyses

Statistical analyses were conducted in R 4.2.3 [61]. Prior to analyses, rTL measures were transformed using the Box–Cox transformation [62] to normality to improve model fits (validated through visualization of diagnostic plots). rTL measures were then transformed to *z*-scores (mean = 0, standard deviation (s.d.) = 1) to improve comparability across studies [63]. 95% confidence intervals of fixed effects were obtained using parametric bootstrapping (*n* = 10 000 iterations). Continuous predictors were mean-centred in order to remove collinearity between their fixed effects and interactions [64]. Final models were achieved through keeping fixed effects regardless of significance but interactions were removed when non-significant [65,66]. In all models detailed, collinearity among predictors was assessed using adjusted generalized variance inflation factor (GVIF) values [67] to account for some predictors having multiple degrees of freedom using a threshold of less than 3.

To explore whether tissue sample storage time and DNA storage time affected rTL, we constructed a linear mixed-effect model (LME) with rTL as the response variable with sample storage time (days), DNA storage time (days), and age of individuals (years) included as fixed effects. Individual identity (accounting for multiple rTL measurements from individuals), matriline (to account for potential family and/or genetic effects) and qPCR plate (accounting for technical variance) were fitted as random effects. We tested whether quadratic terms of both storage time variables significantly improved model fit. Within-individual repeatability (across multiple samples from the same individual taken at different time points) was calculated using the R package *rptR* [68]. Within-individual repeatability was calculated by dividing the between-individual variance in rTL by the total variance of the model after accounting for fixed effects. The model included rTL fitted as a response variable and age, sex, and year of sample as fixed effects, with individual identity and qPCR plate as random effects with 1000 bootstrap iterations. Visual depiction of the data shows patterns of decreases and increases across hibernation sampling sessions (electronic supplementary material, figure S1). Following [69], sensitivity analyses were conducted in a Bayesian framework (MCMCglmm [70]) to compare these within-individual changes to variance among technical replicates (triplicates among plates; see electronic supplementary material, for further details).

#### (i) Factors influencing hibernation relative telomere length

Mixed-effect models were run to explore rTL within hibernation and across winters (see electronic supplementary material, table S1, for a complete list of model variables). An LME was fitted with rTL from all individuals (including individuals sampled only once) as the response variable and sex, hibernation sampling session, hibernation year, and body condition (body mass/forearm length) as fixed effects. Age was examined using a within-centring approach [71,72]. Average age was calculated as the average of all ages of which an individual has an rTL measure, and delta age was calculated as the difference between age at sampling and the individual's average age. Both age covariates were included to discriminate within-individual patterns (delta age) and between-individual patterns (average age). Plausible two-way interactions between fixed effects were also tested including if poor body condition (an indication of energy reserves) at different hibernation sampling sessions impacted telomere length, if ageing patterns differed with sex and if the age structure of individuals sampled remained similar over the hibernation seasons. All continuous predictors were mean-centred and individual identity, matriline, and qPCR plate were included as random effects. To test if between- and within-individual age effects were significantly different (i.e. selective disappearance), we reran the aforementioned model with age (in years) and average age as age covariates. Here, age in years represents the within-individual effect and average age quantifies the difference between within- and between-individual effects [72]. If the average age term is significant in this model, the slopes between and within individuals differ, suggesting selective disappearance/appearance of individuals.

Visual inspection of raw rTL data (electronic supplementary material, figure S1) suggests patterns of increases and decreases across the hibernation season. To explore these patterns, we investigated telomerase (telomerase reverse transcriptase (TERT)) expression in *R. ferrumequinum* using a publicly available wing transcriptome dataset obtained from individuals caught during hibernation in another population sampled in the Jilin Province of northeast China [73]. *Rhinolophus ferrumequinum* from this province experience similar climatic conditions as individuals in this study and undertake winter torpor on a similar timescale [74]. Individuals sampled in [73] were caught in October, December, April and May 2020–2021. Raw RNAseq reads obtained from [73] underwent initial quality control using Trim Galore! v0.6.10 [75]. This removed residual Illumina adaptors and filtered out reads with low-quality bases (Phred quality score less than 20) with default settings for paired-end data. Read quality for the trimmed data was assessed using FastQC [76] and MultiQC [77]. Reads were then mapped to the *R. ferrumequinum* reference genome [78] using STAR v2.7.10 [79]. Gene quantification was quantified using featureCounts v2.0.3 [80] with count normalization and differential gene analysis achieved using the R package *DESeq2* [81].

#### (ii) Effects of weather on relative telomere length change during hibernation

As year effects were detected, analyses were undertaken to explore drivers of telomere variation when considering winter foraging (foraging is expected in warm and dry weather). Within-individual change in rTL was examined using individuals sampled more than once within years. Individuals caught once or across winters were removed leaving 214 measures from 90 individuals (for further details, see electronic supplementary material, table S2). Meteorological data for Gloucestershire (mean daily temperature (°C) and cumulative daily rainfall (mm) were extracted from the Met Office Integrated Data Archive System (MIDAS) [82] from two weather stations active during the sampling sessions and closest to the hibernacula sites (distances: 5.03 km and 10.1 km, mean distance: 7.57 km).

The effects of these weather variables on rTL during hibernation were analysed using a sliding window approach with the *climwin* package [83,84]. This approach identifies the optimal time windows for environmental variables preceding rTL measurements that best explain variation in rTL. The AICc of all models are ranked against a baseline model containing no weather variables with the best-supported model selected based on its *Δ*AICc. We investigated relative sliding windows for both weather variables independently in increments of days, backdating 100 days from each hibernation sampling session. This allowed consideration of short-term weather conditions and late summer/early autumn conditions where mature female bats build up fat reserves prior to or early in the hibernation period. The baseline model for each weather variable was fitted with rTL as the response variable, and sex, average age, delta age, body condition, hibernation sampling session, hibernation year and rTL at first sampling as fixed effects. Individual identity, matriline, and qPCR plate were included as random effects. We considered both linear and quadratic weather effects given visualization of the raw weather data.

To decrease the likelihood of false positive results from multiple comparisons, only windows greater than or equal to 5 days were considered as shorter windows are biologically less plausible and can result in statistical artefacts [83]. The built-in randomization procedure in *climwin* was run to assess the likelihood that the best-observed window occurred by chance. Each model was run on 100 randomized datasets and the distribution of *Δ*AICc from the 100 randomizations was compared to the *Δ*AICc of the observed dataset. We use the metrics *P_Δ_*_AICc_ and *P*_C_ as detailed in [83,84] to assess if windows identified were false positives. If *P_Δ_*_AICc_ was less than 0.05 and *P_C_* < 0.5, we considered these to represent true temperature windows. If both linear and quadratic terms followed these criteria, we chose the model with the lowest ΔAICc. Both weather variables were added into the baseline model to determine significance.

## 3. Results

Accounting for samples that failed DNA extraction and those missing morphometric measurements, the final dataset comprised 388 rTL measures from 223 individuals (203 individuals of known age and 20 individuals of estimated minimum age). These represented 118 females and 105 males with an age range of 0 (first year of life) to 19 years.

### (a) Sample storage duration and sensitivity analyses

In testing for storage effects, quadratic terms of both storage terms did not significantly improve model fit (LRT: *χ*^2^ = 2.196, *p* = 0.334). Sample storage time and DNA storage time were not associated with rTL (*β*_Sample storage time_: 0.0003, 95% CI [−0.0001, 0.0006]; *β*_DNA storage time_: −0.0031, 95% CI [−0.0086, 0.0025]; electronic supplementary material, table S3) and were excluded from subsequent models. Intra-individual repeatability of rTL calculated from individuals with multiple measures of rTL was 0.093 (95% CI [0.005, 0.198]). Sensitivity analyses show that the random effect estimate of individual identity explained more variance in rTL among technical replicates (0.0246; 95% CI [0.0178, 0.0316]) than changes between biological replicates (0.0017; 95% CI [0.0004, 0.0032]; electronic supplementary material). This pattern remained when data were split into groups with positive or negative changes with no overlap of 95% CI for random effect estimates between technical and biological replicates (electronic supplementary material). This indicates changes in rTL are not due to measurement error alone.

### (b) Variation in telomere length

**Figure 1.**
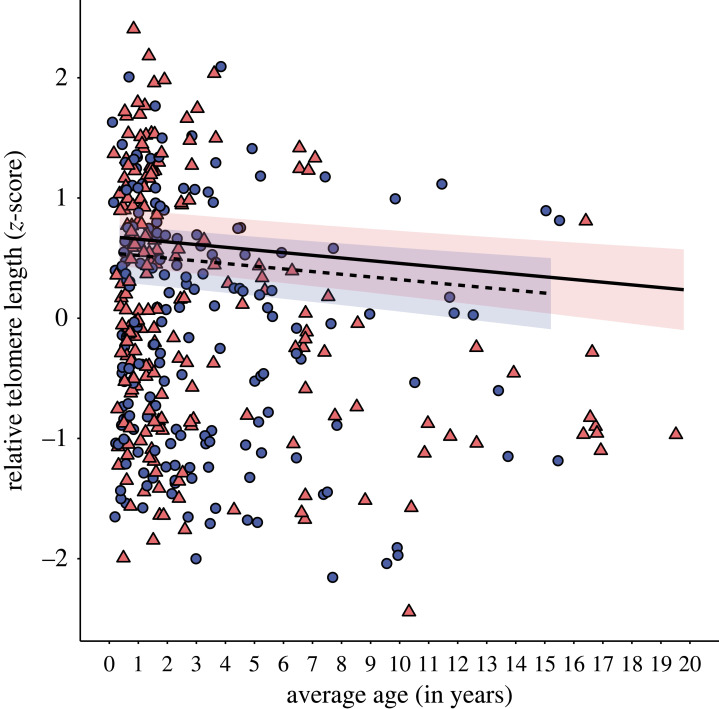
Relative telomere length (rTL) in relation to average age (between-individuals) and sex in hibernating *Rhinolophus ferrumequinum.* Points show rTL data (*z*-scores) with fitted lines based on model estimates with 95% confidence intervals. Points are colour coded by sex (females, pink triangles; males, blue circles). Older *R. ferrumequinum* had shorter telomeres than their younger counterparts with males displaying on average shorter rTL compared with females.

rTL appears highly dynamic with initial decreases across early hibernation sampling sessions (sessions 1–2) with an indication of increases later in hibernation (sessions 3–5; electronic supplementary material, figure S1). When we consider all samples, rTL showed a negative relationship with average age with older individuals displaying shorter telomeres but a similar relationship was not seen within individuals (table 1 and figure 1). For 20 individuals out of the 223 individuals with 29 measures of rTL between them, only minimum age was known as bats were ringed as adults. To avoid potential bias in cross-sectional analyses, the model was re-run without these individuals and this did not change the findings (electronic supplementary material, table S4). Males displayed marginally shorter telomeres than females on average (table 1 and figure 1) but age patterns were not different between the sexes (average age × sex: $\chi_{1}^{2}=0.001$, *p* = 0.974; delta age × sex: $\chi_{1}^{2}=0.441$, *p* = 0.508). Age structure did not differ across the hibernation years (average age × hibernation year: $\chi_{2}^{2}=0.600$, *p* = 0.550). In testing for selective disappearance, no significant difference was found between the slopes of between individuals and within-individual age effects (*β*_Average age_; 95% CI [−0.120, 0.083]; electronic supplementary material, table S5).

**Table 1.** Summary of effects from LME of different predictor variables on hibernation rTL in greater horseshoe bats. Significant values (95% confidence intervals not overlapping zero) are shown in bold. For sex, hibernation year, and hibernation sampling session, the reference categories are female, session 1 and 2015/2016 (s.e., standard error). Estimates for continuous variables are centred. 95% confidence intervals (CI) were obtained from 10 000 iterations of parametric bootstrapping. Random effects variance: individual identity (6.429 × 10^−2^), matriline (8.221 × 10^−3^), qPCR plate (2.800 × 10^−1^), residual (2.348 × 10^−1^).

response variable: rTL	*β*	s.e.	95% CI
*fixed effects* (*n* *=* *388 samples; N* *=* *223 individuals*)			
(intercept)	0.609	0.243	0.133, 1.090
body condition	2.024	1.557	−1.010, 5.059
*sex* (*female*)			
male	−0.135	0.066	**−0.264, −0.005**
average age	−0.022	0.011	**−0.044, −0.001**
delta age	0.007	0.050	−0.091, 0.107
*hibernation year* (*2015/2016*)			
2017/2018	−0.095	0.181	−0.452, 0.271
2019/2020	−1.579	0.274	**−2.118, −1.026**
*hibernation sampling session* (*session 1*)			
session 2	0.294	0.227	−0.162, 0.754
session 3	0.159	0.212	−0.255, 0.581
session 4	−0.213	0.279	−0.762, 0.344
session 5	−0.059	0.226	−0.509, 0.393
*body condition × hibernation sampling session*			
session 2	−1.895	2.172	−6.173, 2.376
session 3	−3.003	2.557	−7.995, 2.047
session 4	−10.128	3.554	**−17.138, −3**.**031**
session 5	−1.288	5.981	−12.715, 10.808

**Figure 2.**
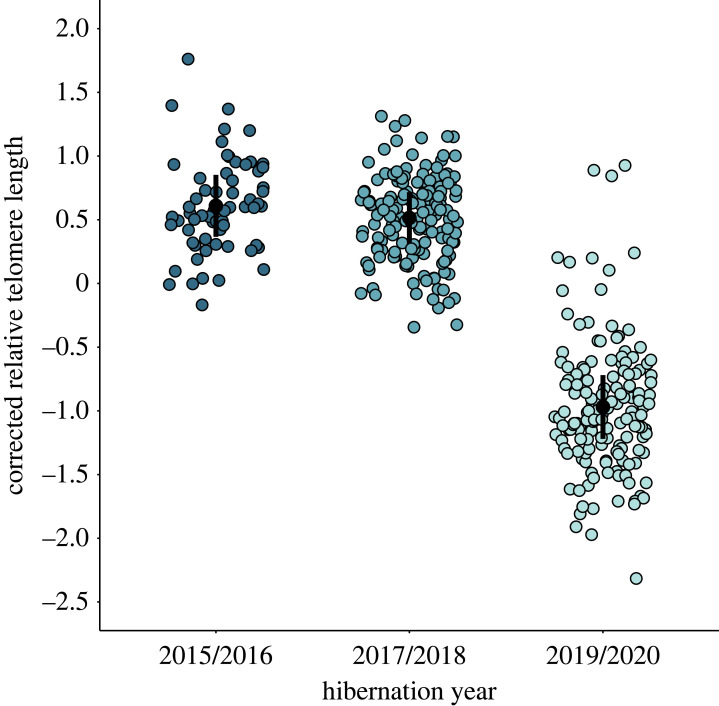
Estimated marginal means (± s.e., standard error) for rTL and model corrected residual rTL of *Rhinolophus ferrumequinum* in different hibernation years. *Rhinolophus ferrumequinum* individuals had significantly lower rTL in 2019/2020 compared to preceding years. Bars represent model predicted values (table 1) corrected for other model covariates and factors at their reference category. Points are residual rTL (corrected for other model predictors) and jittered to show data.

Individuals had significantly shorter telomeres in 2019/2020 compared to previous winters (table 1 and figure 2). The overall general pattern of hibernation rTL did not differ significantly between hibernation sampling sessions (table 2). Individuals in poorer body condition have shorter telomeres during session 4 of hibernation (LME, *β*_body condition*session 4_; 95% CI [−17.138, −3.031]). At this time (late April–early May), body reserves can be low following hibernation, females are in the early stages of pregnancy and bats may be subject to stress before conditions for feeding improve.

**Table 2.** Summary of linear mixed effect models and parametric bootstrapping explaining average daily temperature on rTL of individuals caught more than once within a hibernation year in *R. ferrumequinum*. qPCR plate, individual identity, and matriline were included as random effects. For sex and hibernation sampling session, the reference categories are female and session 1. Significant values are shown in bold. CI, confidence interval; s.e., standard error. Random effects variance: individual identity (9.378 × 10^−2^), matriline (1.077 × 10^−3^), qPCR plate (2.230 × 10^−1^), residual (2.036 × 10^−1^).

response variable: rTL	*β*	s.e.	95% CI
*fixed effects* (*n* *=* *214 samples; N* *=* *90 individuals*)			
intercept	−2.314	1.105	**−4.528, −0.119**
average age	0.005	0.019	−0.032, 0.042
delta age	0.152	0.104	−0.052, 0.356
BCI	0.150	1.851	−3.643, 3.840
*sex* (*female*)			
male	−0.203	0.099	**−0.393, −0.007**
*hibernation sampling session* (*session 1*)			
session 2	2.915	1.067	**0.812, 5.055**
session 3	3.703	1.249	**1.304, 6.225**
session 4	2.761	0.976	**0.887, 4.724**
session 5	4.314	1.583	**1.192, 7.475**
average daily temperature	−1.807	0.742	**−3.282, −0.345**
average daily temperature^2^	0.140	0.054	**0.033, 0.248**
*hibernation year* (*2015/2016*)			
2017/2018	−0.216	0.276	−0.758, 0.335
2019/2020	−0.011	0.634	−1.277, 1.252

### (c) Weather effects and telomere length increases

**Figure 3.**
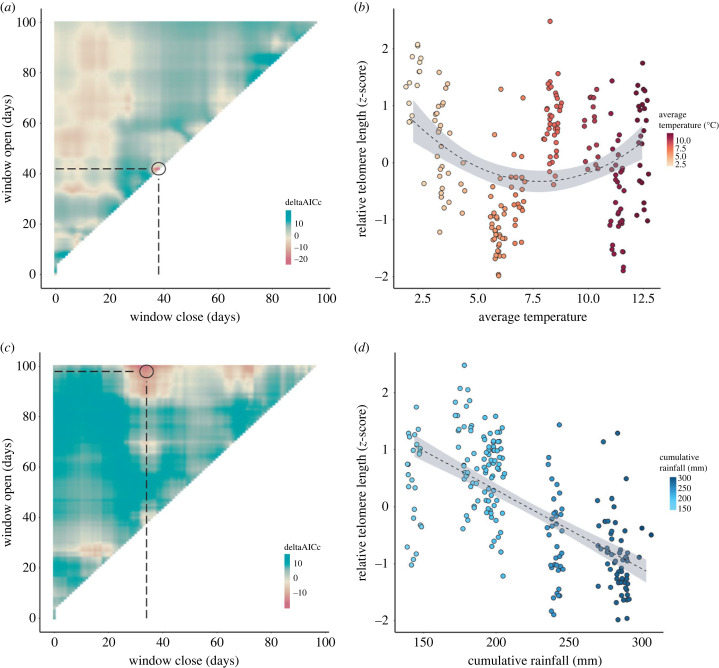
Sliding window analysis of the effects of (*a*) average daily temperature and (*c*) cumulative daily rainfall on relative telomere length (rTL); and rTL as a function of (*b*) average daily temperature and (*d*) cumulative rainfall in their respective time windows. (*a,c*) Delta AICc values represent the differences between the AICc of each climate window model and the baseline model with no climate variable. Larger negative values indicate stronger support for a given climate window. The circle and dotted lines represent the most sensitive time window greater than or equal to 5 days (mean daily temperature (quadratic), 38–42 days; cumulative daily rainfall (linear), 31–100 days). (*b*) In the selected time window (38–42 days prior to sampling), rTL shows an initial decline with increasing temperature with evidence of increases towards higher temperatures; (*d*) while increasing cumulative rainfall (31–100 days prior to sampling) leads to shorter rTL. Fitted lines based on model estimates with 95% confidence intervals.

For tested weather variables in sliding window analyses (electronic supplementary material, table S6), average daily temperature (quadratic) 38–42 days (ΔAICc = −20.37; figure 3*a*) shows a positive quadratic relationship with rTL (figure 3*b* and table 2). Cumulative daily rainfall (linear) 31–100 days (ΔAICc = −16.98; figure 3*c*) prior to all sampling sessions had a negative effect on rTL change (figure 3*d* and table 3). Randomizations show that these signals are unlikely to be false positives with *P_Δ_*_AICc_ being less than 0.05 and *P_C_* < 0.5 for both weather variables (electronic supplementary material, table S6, figure S2). Inclusion of weather variables back into the baseline showed rainfall was collinear with temperature (GVIF = 10.042) and temperature and rainfall were analysed separately.

**Table 3.** Summary of linear mixed effect models and parametric bootstrapping explaining cumulative rainfall on rTL of individuals caught more than once within a hibernation year in *R. ferrumequinum*. qPCR plate, individual identity, and matriline were included as a random effects. For sex and hibernation sampling session, the reference categories are female and session 1. Significant values are shown in bold. CI, confidence interval; s.e., standard error. Random effects variance: individual identity (9.493 × 10^−2^), matriline (9.981 × 10^−3^), qPCR plate (2.325 × 10^−1^), residual (2.055 × 10^−1^).

response variable: rtl	*β*	s.e.	95% CI
*fixed effects* (*n* *=* *214 samples; N* *=* *90 individuals*)			
intercept	0.209	0.398	−0.583, 1.009
average age	0.005	0.019	−0.034, 0.043
delta age	0.152	0.104	−0.047, 0.357
BCI	0.050	1.858	−3.687, 3.727
*sex* (*female*)			
male	−0.203	0.100	**−0.396, −0.008**
*hibernation sampling session* (*session 1*)			
session 2	0.292	0.324	−0.364, 0.930
session 3	0.551	0.334	−0.117, 1.205
session 4	0.658	0.345	−0.021, 1.344
session 5	0.264	0.302	−0.333, 0.872
cumulative daily rainfall	−0.010	0.004	**−0.018, −0.017**
*hibernation year* (*2015/2016*)			
2017/2018	−0.139	0.238	−0.620, 0.341
2019/2020	−0.769	0.465	−1.696, 0.164

**Figure 4.**
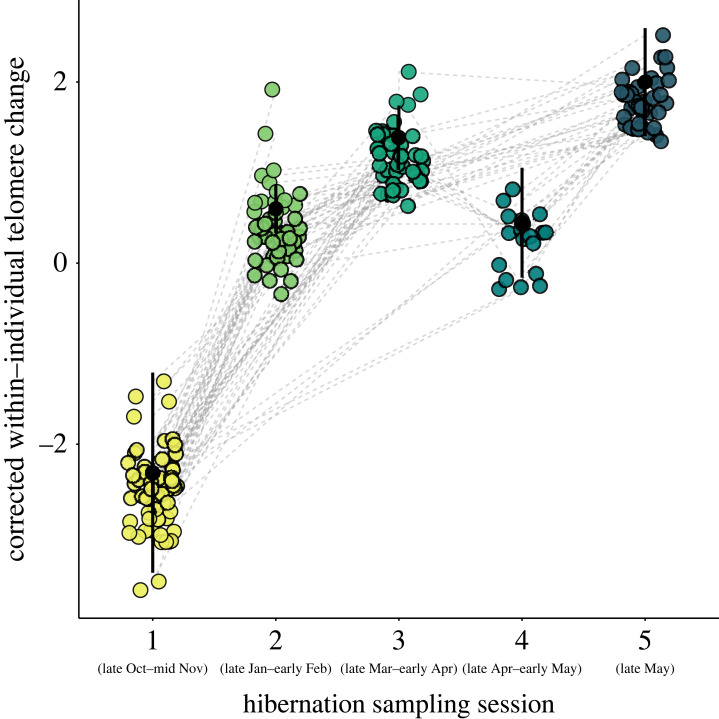
Estimated marginal means (± s.e., standard error) for telomere length between different hibernation sampling sessions from 90 *Rhinolophus ferrumequinum* individuals sampled more than once within hibernation years. Increases in telomere length are observed across the hibernation sampling sessions (table 2) in *R. ferrumequinum*. Bars represent model predicted values (table 2). Grey dashed lines connect repeat samples from the same individual. Points are residual rTL (corrected for other model predictors).

rTL displayed an initial decline with temperature with evidence of increases at higher temperatures (figure 3*b* and table 2), potentially reflecting short-term benefits of foraging during mild conditions. By contrast, higher cumulative rainfall resulted in shorter telomeres as foraging is expected to be reduced during heavy rain conditions (figure 3*d* and table 3). When hibernation year was included in models, it became non-significant while weather variables remained significant (tables 2 and 3), indicating weather potentially explains the observed yearly variation. However when accounting for weather condition impacts, increases are observed across sampling sessions within hibernation years (figure 4), with 46 out of the 90 individuals (51% of samples) showing an increase in rTL between consecutive measures. Using a publicly available wing transcriptome dataset available from the same species in the Jilin Province (China) hibernating in similar conditions, telomerase (TERT) was quantified (electronic supplementary material, figure S3). TERT expression was found in the wing transcriptome and increased over the hibernation period (electronic supplementary material, figure S3), suggesting that lengthening and/or maintenance of telomeres in this species may be maintained by telomerase.

## 4. Discussion

Telomere lengths did not significantly differ between hibernation sampling sessions when we considered all *R. ferrumequinum* individuals caught during hibernation. There was a between-individual effect of age with between- and within-individual slopes not differing, suggesting no evidence of selective disappearance. Strong year effects were found, with shorter telomeres linked with higher rainfall and varying patterns of temperature. When accounting for weather conditions, within-individual increases were observed in over half of the individuals caught more than once within a hibernation year. These patterns could potentially be attributed to increased telomerase expression during hibernation in this species, though this needs further validation in this population.

Previous cross-sectional analyses showed a linear decrease of rTL with age in *R. ferrumequinum* rTL (0–24 years of age [45]), with a between-individual decline of rTL with age recovered in this study. Within-individual change of rTL was non-significant with no significant difference with between- and within-individual slopes. While no selective disappearance patterns were found, a lack of within-individual change could potentially reflect the structure of our data. Sampling occurred over 3 years (in contrast to *R. ferrumequinum*'s long maximum lifespan), restricting potential for multiple rTL measures from some individuals. This is likely insufficient in detecting much within-individual change, a factor that future studies should consider. Male *R. ferrumequinum* on average had shorter rTL, which did not vary with age. This may reflect the different costs that the sexes experience at different times of the year (e.g. territorial males carry out intensive mating activities from September to November each year, resulting in depletion of their fat stores [52]). Adult male *R. ferrumequinum* need to arouse more during hibernation to exploit foraging opportunities after the intense mating period and for further mating events [52]. Young of the year also need to forage in winter as they continue being active well into October, especially if born late. These two groups are most likely to die if winters are severely cold [48,49]. This provides an interesting avenue in exploring the effects of arousals on telomere lengths in bats, and if sex-specific hibernation strategies occur.

Hibernation rTL differed between hibernation years, with individuals sampled in the 2019/2020 season having significantly lower rTL than previous years. Expansion of analyses focusing on weather variables showed that wet conditions and suboptimal temperatures for foraging led to shorter telomeres. This influence begins from mid-August where *R. ferrumequinum* typically begin to mate, peaking in October, by which time mature females have deposited large levels of fat for hibernation, but mature males have not [49]. Most *R. ferrumequinum* select hibernacula that fluctuate in ambient temperature in order to suitably time arousals from torpor to warmer nights [11,47,54]. This is linked to foraging through increased abundance of aerial insect prey above 10°C [85]. However, arousals can cost bats up to 80–90% of their energy stores in the absence of winter foraging [10]. Telomere lengths showed an initial decline with average temperature followed by increases at higher temperatures. Studies in dormice (Gliridae) show individuals undergoing torpor at higher temperatures displayed less telomere attrition due to arousals than those hibernating at colder temperatures [30]. This is likely due to rewarming from higher temperatures being less costly than colder temperatures, with increased food availability shown also to compensate at higher temperatures [30,86]. A potential explanation for the shorter rTL in 2019/2020 involves individuals arousing to feed given the higher temperatures but suffering from poor, or no foraging success due to the associated heavy rainfall. The 2019/2020 hibernation period was one of the warmest and wettest winters on record in the UK [87]. Wet and windy conditions have been correlated with reduced food consumption throughout summer (April–September) in females from this population, showing the effect of weather on bat foraging capabilities [88]. Heavy rainfall is likely to impose additional costs in bats, on top of the costs of arousals from torpor. It affects bat thermoregulation, leading to increased energetic costs of flight [89]. Additionally, there is likely little to gain as aerial insect abundance is reduced, even in warm temperatures [89]. Hibernation rTL could provide insight into seasonal effects and how bats might cope with predicted increases in winter temperature and rainfall under future climate change scenarios [87].

Interestingly, while there are yearly effects on rTL, within-individual increases and/or maintenance of telomere length in some individuals occurred during hibernation after accounting for weather conditions. Individuals showed low repeatability in rTL (9.3%), similar to previous estimates in other mammalian wild populations (e.g. 13% [90], 6.5% [46], 2.2% [69]) but lower than the average value for qPCR studies and those carried out using the terminal restriction fragment (TRF) method [91]. This is often attributed to qPCR having a higher susceptibility to measurement error than TRF, while TRF also shows high intra-individual repeatability and high heritability estimates in contrast to qPCR [91,92]. In addition, qPCR measures all telomeric sequences, including interstitial telomeres in contrast to in-gel TRF methods [93]. Samples in this study were randomized on qPCR plates along with other samples collected at different time points as part of this long-term project. While this removes confounding effects between year of samples and batch of samples, randomization can create variation between clusters, potentially adding a source of error behind measurements [94]. While often attributed to measurement error, within-individual increases in this study cannot be fully explained by measurement error alone, similar to other studies [69,95]. While interstitial telomeric repeats may account for such patterns, interstitial telomeres are likely to remain more consistent over lifetimes, with little evidence they are susceptible to environmental effects as seen in terminal telomeric repeats [96,97]. Lengthening could be potentially due to cell turnover rate in wing tissue, but more studies are needed to estimate this rate in bats [98]. While mechanistic reasons for telomere lengthening are possible (see below), patterns of telomere lengthening should be interpreted with caution.

Analyses of wing transcriptomic data from the same species in another population caught and sampled over the hibernation year show that telomerase is expressed in the wing, with telomerase expression increasing over the course of hibernation. Increases or maintenance of telomere length have also been observed during torpor in different hibernators [29–31,33] but telomerase activity was not explored simultaneously in these studies. Telomerase expression has only been investigated in two other bat species [45,99]. Transcriptome analysis of whole blood and primary wing-based fibroblast cultures showed no TERT expression in *M. myotis* [45]. By contrast, higher expression of telomerase was shown to occur during hibernation across several tissues when compared with an active individual in a closely related species to *R. ferrumequinum*, the great roundleaf bat (*Hipposideros armiger*) [98]. Hibernation is associated with slower epigenetic ageing in big brown bats (*Eptesicus fuscus*) [100]. Increases in telomere length during torpor periods could indicate a form of somatic maintenance in *R. ferrumequinum* compared to active periods in the life cycle (e.g. early life growth) where telomere lengths decrease rapidly over short timescales [98]. Yet, telomerase can have negative effects such as its role in proliferating cancers [101]. Given low cancer incidences in bats [102], telomerase expression must be tightly regulated to ensure that enough is produced to maintain telomere length without driving malignancy. However, little is known about general patterns of telomerase activity in bats, with varying patterns of expression from the few bats studied [28,45] in addition to variation in torpor strategies. Analysis of telomerase expression in this *R. ferrumequinum* population will provide further insight as well as determining how telomerase expression varies across populations. In addition, comparative analyses could determine how telomeres and telomerase expression vary with torpor strategies in bats as well as telomere dynamics during active periods in the life cycle.

This research is the first to quantify telomere length dynamics during hibernation in a long-lived flying mammal, *R. ferrumequinum*, which differs in thermoregulation strategies, fat storage, and use of hibernacula compared with the more extensively studied rodents and carnivores. Here, rTL reflected yearly weather conditions, with evidence of within-individual increases of rTL over the hibernation year in over half of the individuals caught more than once. Increases seen are likely due to telomerase expression but this needs to be explored in further studies. Future work should explore how torpor bouts and their durations influence telomere length as well as measuring telomerase activity to further understand patterns of telomere maintenance during hibernation in bats.

## Data Availability

Data are available from the Dryad Digital Repository: https://doi.org/10.5061/dryad.g79cnp5vw [103]. Supplementary material is available online [104].
